# A Survey of Mycoviral Infection in *Fusarium* spp. Isolated from Maize and Sorghum in Argentina Identifies the First Mycovirus from *Fusarium verticillioides*

**DOI:** 10.3390/v12101161

**Published:** 2020-10-14

**Authors:** Andrés Gustavo Jacquat, Martín Gustavo Theumer, María Carmen Cañizares, Humberto Julio Debat, Juliana Iglesias, María Dolores García Pedrajas, José Sebastián Dambolena

**Affiliations:** 1 Facultad de Ciencias Exactas Físicas y Naturales (FCEFyN), Universidad Nacional de Córdoba (UNC), Córdoba 5000, Argentina; agjacquat@imbiv.unc.edu.ar; 2 Instituto Multidisciplinario de Biología Vegetal (IMBIV), Consejo Nacional de Investigaciones Científicas y Técnicas (CONICET), Avenida Vélez Sarsfield 1611, Córdoba X5016GCA, Argentina; 3 Departamento de Bioquímica Clínica, Facultad de Ciencias Químicas (FCQ), Universidad Nacional de Córdoba (UNC), Córdoba 5000, Argentina; mgtheumer@fcq.unc.edu.ar; 4 Centro de Investigaciones en Bioquímica Clínica e Inmunología (CIBICI), Consejo Nacional de Investigaciones Científicas y Técnicas (CONICET), Haya de la Torre y Medina Allende—Ciudad Universitaria, Córdoba X5000HUA, Argentina; 5 Instituto de Hortofruticultura Subtropical y Mediterránea “La Mayora”, Universidad de Málaga, Consejo Superior de Investigaciones Científicas (IHSM—UMA—CSIC), Estación Experimental “La Mayora”, Avenida Dr. Wienberg s/n, 29750 Algarrobo-Costa, Málaga, Spain; carmen.canizares@eelm.csic.es; 6 Instituto de Patología Vegetal, Centro de Investigaciones Agropecuarias, Instituto Nacional de Tecnología Agropecuaria (IPAVE—CIAP—INTA), Camino 60 Cuadras Km 5.5, Córdoba X5020ICA, Argentina; debat.humberto@inta.gob.ar; 7 Estación Experimental Pergamino, (EEA) INTA Pergamino, Universidad Nacional Noroeste (UNNOBA), Pergamino (Buenos Aires) B2700, Argentina; iglesias.juliana@inta.gob.ar

**Keywords:** maize, sorghum, *Fusarium verticillioides*, *Fusarium andiyazi*, mycovirus, mitovirus FvMV1, FaMV1-162

## Abstract

Mycoviruses appear to be widespread in *Fusarium* species worldwide. The aim of this work was to identify mycoviral infections in *Fusarium* spp., isolated from maize and sorghum grown in Argentina, and to estimate their potential effects on the pathogenicity and toxigenesis of the host fungus towards maize. Mycoviruses were identified in 2 out of 105 isolates analyzed; *Fusarium verticillioides* strain Sec505 and *Fusarium andiyazi* strain 162. They were characterized as members of the genus *Mitovirus* by high-throughput sequencing and sequence analysis. The *F. verticillioides* mitovirus was a novel mycovirus whereas the *F. andiyazi* mitovirus was found to be a new strain of a previously identified mitovirus. We have named these mitoviruses, Fusarium verticillioides mitovirus 1 (FvMV1) and Fusarium andiyazi mitovirus 1 strain 162 (FaMV1-162). To our knowledge, FvMV1 is the first mycovirus reported as naturally infecting *F. verticillioides*, the major causal agent of ear rot and fumonisin producer in corn. Both mitoviruses exhibited 100% vertical transmission rate to microconidia. The Fa162 strain infected with FaMV1-162 did not show phenotypic alterations. In contract, *F. verticillioides* Sec505 infected with FvMV1 showed increased virulence as well as microconidia and fumonisin-B1 production, compared with two uninfected strains. These results suggest that FvMV1 could have a role in modulating *F. verticillioides* pathogenicity and toxin production worth further exploring.

## 1. Introduction

Mycoviruses are viruses that infect and replicate in fungal cells [1,2,3]. A great diversity of mycoviruses has been identified in a wide variety of fungal species [4,5]. The majority of mycoviruses have double-stranded RNA (dsRNA) genomes or positive sense single-stranded RNA (+ssRNA) genomes with dsRNA replicative intermediates [5,6]. However, mycoviruses with +ssRNA genomes with reverse transcriptase (+ssRNA-RT), negative sense ssRNA (-ssRNA) genomes, and DNA mycoviruses have also been reported [4]. Mycovirus genomes can be protected or not by a protein capsid [4,7]. According to the International Virus Taxonomy Committee (talk.ictvonline.org/taxonomy/), mycoviruses have been taxonomically grouped into 17 formal families. Mycoviruses with dsRNA genomes have been classified in 7 families (*Megabirnaviridae*, *Partitiviridae*, *Quadriviridae*, *Reoviridae*, *Totiviridae*, *Chrysoviridae*, and *Endornaviridae*) and one kingdom (*Orthornavirae*). Families *Alphaflexiviridae*, *Barnaviridae*, *Gammaflexiviridae*, *Hypoviridae*, *Tymoviridae, Mitoviridae*, and *Narnaviridae* have +ssRNA genomes. Families *Mymonaviridae* and *Metaviridae* are comprised of mycoviruses with –ssRNA and +ssRNA-RT genomes, respectively. Mycoviruses with single-stranded DNA (ssDNA) genomes have been classified in the family *Genomoviridae* [8]. New mycoviruses are constantly reported, and many still remain unclassified [6] or are classified into newly proposed families, such as *Fusariviridae* [9,10], *Tetramycoviridae* [11], *Polymycoviridae* [12], *Alternaviridae* [13], and *Yadokariviridae* [14].

Mycoviruses are isolated from fungi with very diverse lifestyles, and their infections are often cryptic, that is, no discernable phenotypic alterations are observed in the host [3,4,5]. However, in phytopathogenic fungi, there are mycoviruses that cause hypovirulence (reduced virulence) [15]. Hence, the study of mycoviruses from plant pathogens receives considerable attention due to their potential application as tools for the biocontrol of crop diseases [16,17,18,19]. The vast majority of mycoviruses lack an extracellular route of horizontal transmission, but they are efficiently transmitted by hyphal anastomosis between vegetatively compatible strains of the same species [3,20]. This feature allows transmission of the hypovirulence-inducing mycovirus to natural isolates of the pathogen, upon introduction of infected fungal strains in the environment, while preventing its horizontal jump to other fungal species and organisms. Mycoviruses have, therefore, the potential for being efficient and very safe biological control agents to be used in the field [19,21]. *Chryphonectria hypovirus 1* (CHV1) was the first mycovirus reported to cause hypovirulence in its host, the ascomycete fungus *Chryphonectria parasitica*, the causal agent of chestnut blight [22,23]. A decrease in the incidence of chestnut blight in Europe was found to be associated with the natural spread of CHV1 in the populations of the pathogen [24]. This led to the implementation of a chestnut blight control program based on the use of CHV1, which is currently in effect in Europe [24,25,26]. The hypovirulence induced by CHV1 in *C. parasitica* is associated with other phenotypic alterations, such as a reduction in conidiation, pigmentation, and vegetative growth [15,23]. CHV1 is the type species of the genus *Hypovirus*, in the family *Hypoviridae* of capsidless mycoviruses with +ssRNA genomes. Currently, the ability of mycoviruses to induce hypovirulence and other host phenotypic alterations, from a great variety of viral families, has been reported [19]. Several mycoviruses, whose infection results, by contrast, in increased virulence towards the host plant have also been identified [27,28,29]. These mycoviruses are also of interest in plant pathology, since the characterization of their interaction with the host can provide new insights into the molecular regulation of virulence in fungi.

*Fusarium* is a genus of filamentous fungi of the phylum Ascomycota, with recognized ability to cause disease. These fusariosis can result in symptoms like blights, rots, cankers, and wilts, in any organ and tissue, and affect a wide range of crops, including forest, cereal, and horticultural crops [30,31,32,33], causing significant economic losses [34,35]. In addition, some *Fusarium* species have the capacity to produce mycotoxins, mainly fumonisins, being the B series (FBs), zearalenone, trichothecenes, and deoxynivalenol (DON), the ones that represent a considerable problem due to their toxicological implications in humans and farm animals [36,37]. In extensive cropping systems around the world, the use of synthetic fungicides is essential to prevent or cure fungal diseases, as part of an integrated disease management approach [38,39]. However, treatment with synthetic fungicides has frequently limited or no effect in the control of *Fusarium* diseases [40,41]. Moreover, the massive use of synthetic agrochemicals has negative impacts on the environment [42,43,44], public health [45,46], and food security [47,48]. Therefore, there is a need to explore new management strategies that are both efficient and environmentally friendly, such as those relying on biological control agents [44,49,50]. As in other major plant pathogens, the use of mycovirus-mediated hypovirulence has been proposed as a potential method for the biological control of *Fusarium* species [51]. The search for mycoviruses that attenuate fungal virulence, and have therefore potential as biological control agents [16], generally focuses on the identification and characterization of those naturally infecting the pathogen to be controlled [18,19]. To date, mycoviruses have been isolated from a variety of *Fusarium* species [51,52]. These mycoviruses belong to a wide range of viral families with dsRNA, +ssRNA, and −ssRNA genomes (recently reviewed by Li et al. [52]). However, only a limited number of them have been associated with the induction of phenotypic alterations in the host fungus. Mycoviruses associated with the attenuation of virulence in *Fusarium* species include *Fusarium graminearum chrysovirus* (FgV-China 9) [53] and *Fusarium oxysporum chrysovirus 1* (FodV1) [54,55], both included within the family *Chrysoviridae*, Fusarium graminearum hypovirus 2 (FgHV2), which is a putative member of the family *Hypoviridae* [56], and Fusarium graminearum virus 1 (FgV1) [57,58].

Several of the mycoviruses identified in *Fusarium*, in addition to causing hypovirulence and alterations in vegetative growth, affect mycotoxin production [59]. For instance, fungal strains infected with FgHV2 exhibit reduced DON production, whereas FgV1 infection affects trichothecene mycotoxin production [56,58]. These results show that mycoviruses may have potential to control both virulence and mycotoxins. The ability of some mycoviruses to either repress or induce mycotoxin production has been reported in other groups of fungi, including plant pathogens and species with other life styles. For example, the increase in pathogenicity observed in *Alternaria alternata* strains with high titer of the chrysovirus AaCV1 was associated with an overproduction of AK-toxin [28]. *Tolypocladium cylindrosporum* is a fumonisin-producing entomopathogenic fungus, which is also isolated as an endophyte from grasses. A survey of endophytic strains of this fungus showed a significant increase in fumonisin B_2_ production in mycovirus-infected strains [60]. Similarly, Nerva et al. [61] found that *Aspergillus ochraceus virus* (AoV), a partitivirus widespread in *Aspergillus ochraceus*, can cause an overproduction of the mycotoxin ochratoxin A (OTA). According to these results, there is a relationship between mycovirus infection and mycotoxins worth exploring, since it might have implications in their production in field conditions and/or be used to shed further light into the molecular mechanisms that regulate their synthesis.

*Fusarium verticillioides* is a fungus known to infect maize, sorghum, and rice worldwide [62,63,64]. In corn, colonization pathways include different routes of entry such as roots, stems, floral stigma, and grains [64]. Upon plant colonization, it may behave as an endophyte not causing symptoms [65], or become a parasitic agent generating different symptoms, such as seedling blight, stalk rot in adult plants, and ear rot [63,64]. Moreover, *F. verticillioides* is the major FB producer (FB_1_, FB_2_, FB_3_, and FB_4_) in grains [66,67], causing both significant economic losses and toxic effects on farm animals and humans [68,69,70,71]. Fungicides are not efficient in managing *F. verticillioides* disease symptoms and fumonisin contamination of maize kernels, and more efficient and environmentally-friendly approaches have to be found to control this fungus and its mycotoxins. In contrast to other *Fusarium* species, including major pathogens of cereal crops, there were no reports on mycoviruses isolated from *F. verticillioides*. The objective of the present study was to identify mycoviruses infecting *F. verticillioides* and to characterize their potential effects on the host. For that reason, we conducted a survey of mycoviral infection in a collection of *Fusarium* isolates obtained from maize and sorghum grown in Argentina. This analysis led to the identification of Fusarium verticillioides mitovirus 1 (FvMV1) as the first mycovirus known to naturally infect *F. verticillioides*. Initial data suggests that FvMV1 infection might result in increased virulence and fumonisin production. Additionally, in an *F. andiyazi* isolate from sorghum, we identified a new virus strain of Fusarium andiyazi mitovirus 1 (FaMV1-162). Unlike FvMV1, preliminary studies indicate that FaMV1-162 has no effect on its host phenotype.

## 2. Materials and Methods

### 2.1. Fungal Isolates

A total of 99 strains of *Fusarium* spp. were isolated from maize kernels collected from different areas of Argentina. Maize grains were disinfected by immersion in 5.0% NaClO solution for 1 min, rinsed twice with sterile distilled water, and incubated on potato dextrose agar (PDA) medium (Britania Lab. S.A, CABA, Bs. As. Arg.) at 25 °C, until fungal growth was observed. Monosporic cultures were prepared from fungal colonies with typical morphology, pigmentation, and growth rates of *F. verticillioides* [72]. In a subset of 11 isolates, species ascription was confirmed by PCR using specific primers for *F. verticillioides* PQF5-F: 5′-GAGCCGAGTCAGCAAGGATT-3′ and PQF5-R: 5′-AGGGTTCGTGAGCCAAGGA-3′, as described by Sampietro et al. [73]. The species identity of the mycovirus-infected *Fusarium* isolates and the uninfected *F. verticillioides* and *F. andiyazi* isolates used for phenotypic comparisons was confirmed molecularly by the amplification and sequencing of *beta-tubulin* (*β-TUB*) and *translation elongation factor 1 alpha* (*α-TEF 1*) genes. Primers combinations Tub1 (5′-AACATGCGTGAGATTGTAAGT-3′) and Tub2 (5′-TAGTGACCCTTGGCCCAGTTG-3′), and Ef1 (5′-ATGGGTAAGGARGACAAGAC-3′) and Ef2 (5′-GGARGTACCAGTSATCATG-3′) were used to amplify *ß-TUB* and *α-TEF 1* gene fragments, respectively. Amplified fragments were sequenced by the Sanger method, and subjected to Basic Local Alignment Search Tool (BLAST, blast.ncbi.nlm.nih.gov/Blast.cgi) searches against a The National Center for Biotechnology Information (NCBI, www.ncbi.nlm.nih.gov) *Fusarium* nucleotide collection database. The wild-type mycotoxigenic isolate *F. verticillioides* strain M3125 [74] was provided by Dr. Robert Proctor (United States Department of Agriculture, Agricultural Research Service, National Center for Agricultural Utilization Research, Peoria, IL, USA). Additionally, six *Fusarium spp.* isolates, obtained from sorghum provided by Iglesias, J. (INTA—UNNOBA), were included in our survey of mycovirus infection. These were morphologically identified as *F. andiyazi*.

### 2.2. Detection of dsRNAs in Fusarium Isolates

To test for the presence of large molecules of double-stranded (ds) RNA indicative of viral infection, strains were grown in potato dextrose broth for 96 h at 25 °C. Fungal mycelium was then collected by filtration through Miracloth membranes (EMD Millipore Corp., Burlington, MA, United States) and ground to a fine powder in a mortar and pestle in the presence of liquid nitrogen. Approximately 3.5 g of ground fungal tissue from each isolate was used to purify dsRNAs by chromatography on cellulose (CAS# 9004-34-6; Sigma-Aldrich Corp., Burlington, MA, USA), following the methodology described by Valverde et al. [75]. Nucleic acids were electrophoretically fractionated on a 0.8% agarose gel. When dsRNA bands were observed, their molecular nature was confirmed by digestion with DNase I (La Roche Ltd., Basilea, Suiza) and S1 nuclease (Promega Corp. Madison, WI, USA), which degrade single- and double-stranded DNA, and single-stranded DNA and RNA, respectively.

### 2.3. Next Generation Sequencing of dsRNAs and Data Analysis

To obtain RNA for sequencing, Czapek-Dox Liquid (Oxoid) cultures were prepared from infected strains *F. verticillioides* FvSec505 and *F. andiyazi* Fa162. Fungal tissue was collected by filtration through Miracloth (EMD Millipore Corp., Burlington, MA, USA) membranes and ground in a mortar and pestle in the presence of liquid nitrogen to form a fine powder. Total RNA was extracted and purified using RNeasy Plant Mini Kit (QIAGEN N.V. Hilden, Germany), following the manufacturer’s protocol. To eliminate any trace of DNA, the samples were treated with RQ1 RNase-Free DNase (Promega Corp., Madison, WI, USA) RNA was quantified using a BioSpec-nano (Shimadzu Corp. Nakagyo-ku, Kyoto, Japan) spectrophotometer. The quality of the RNA samples was determined by standard agarose gel electrophoresis (0.8%) in TAE buffer. The samples were depleted of rRNA using the Ribo-Zero kit (Illumina, Inc., San Diego, CA, USA) and subjected to 250~300 bp insert stranded-specific cDNA library construction. The cDNA library was then enriched by PCR and subjected to deep sequencing using Illumina NovaSeq platforms with paired-end 150 bp (PE 150) sequencing strategy. Library construction and deep sequencing were performed by Novogene Corporation Inc. (University of California, Sacramento, CA, USA). Illumina NovaSeq high-throughput sequencing (HTS) of RNA from the *F. verticillioides* Sec505 isolate sample rendered a total of 24,788,316 paired end (PE) 150 nt reads. After trimming and quality filtering using Trim Galore (www.bioinformatics.babraham.ac.uk/projects/trim_galore/), the remaining 24,785,446 PE reads were de novo assembled with Trinity v2.8.6 (https://github.com/trinityrnaseq/trinityrnaseq/wiki), with standard parameters, resulting in 25,440 transcripts (mean length 1851 nt). The obtained contigs were subjected to bulk BLASTx searches (E-value < 1 × 10^−5^) against a viral reference sequence (RefSeq) dataset, from NCBI available at https://ftp.ncbi.nlm.nih.gov/refseq/release/viral/viral.1.protein.faa.gz. Only a single 2468 nt long transcript obtained a significant hit (E-value = 0), sharing 44.48% identity with the RdRp of Alternaria arborescens mitovirus 1 (YP_009270635.1). The virus-like contig was subsequently polished by re-mapping the filtered reads using Bowtie 2 (http://bowtiebio.sourceforge.net/bowtie2/index.shtml), with the very-fast-local preset parameter, which rendered a highly supported (mean coverage = 12,136 X; total virus PE reads = 192,206; virus reads as % of total reads = 0.77%) virus sequence of 2471 nt in length. The HTS of *F. andiyazi* 162 RNA rendered a total of 24,856,719 PE-150 reads. After trimming and quality filtering using Trim Galore, the remaining 24,852,431 PE reads were de novo assembled with Trinity, resulting in 25,451 transcripts (mean length 1617 nt). The obtained contigs were subjected to bulk BLASTx searches (E-value < 1 × 10^−5^) against a NCBI virus proteins refseq database. Only a single 2437 nt long transcript obtained a significant hit (E-value = 0), sharing 49.71% identity with the RdRp of Fusarium poae mitovirus 1 (YP_009272898.1). The virus-like contig was subsequently polished by re-mapping the filtered reads using Bowtie 2, which rendered a highly supported (mean coverage = 28,836 X; total virus PE reads = 466,102; virus reads as % of total reads = 1.87%) virus sequence of 2441 bp. Then, the cured viral sequences were annotated by scanning for Open Reading Frames (ORF). To this end, the nucleotide sequences were imported into ORFinder, as implemented in https://www.ncbi.nlm.nih.gov/orffinder/, with a minimal 150 nt ORF length, and genetic code 4 parameters. Conserved domains of the predicted translated products were searched using the NCBI Conserved Domain Database v3.18 tool (CDD; www.ncbi.nlm.nih.gov/Structure/cdd/wrpsb.cgi). Potential secondary structure predictions of the predicted UTR regions and free energy (dG) estimations were conducted using MFOLD software (MFOLDROOT: http://unafold.rna.albany.edu/). The protein deduced molecular weights were estimated in the online software: https://web.expasy.org/peptide_mass/.

The virus sequences characterized in this study are available at NCBI-GenBank, under accession numbers MT506024 (FvMV1) and MT506025 (FaMV1-162). 

### 2.4. Phylogenetic Analyses

Phylogenetic insights were generated following the descriptions of Nibert et al. [76] and Yao et al. [77], with some modifications. Multiple sequence alignments of RdRp sequences were performed by optimized automatic adjustment using MAFTT version 7 [78] at http://mafft.cbrc.jp/alignment/server/ (iterative refinement methods: L-INS-i strategy). The phylogenetic tree was constructed using the MEGA X version 10.1.5 software [79]. Abbreviated mitoviruses names and NCBI accession numbers (partial sequences were excluded): AaMV1 (QDB74990.1), Aarb.MV1 (YP_009270635.1), BcMV1 (YP_002284334.2), BcMV3 (YP_009182161.1), BcMV4 (CEZ26303.1), BsMV1 (AHY03257.1), CcMV1b (AY328477.1), Cfal.MV1 (MK279482.1), Cfru.MV1 (LC497424.1), CpMV1 (NP_660174.1), Enec.MV1 (YP_009465715.1), Enec.MV2 (ATS94399.1), Enec.MV3 (YP_009465717.1), EnMV1 (QDB74989.1), FaMV1-DH06 (QIQ28423.1), FaMV2 (MN295970.1), FbMV1 (BBG56024.1), FcMV1 (AHI43533.1), FcMV2.1 (AHI43534.1), FcoMV1 (YP_009126873.1), FgMV1 (YP_009126872.1), FodMV1 (QIC51112.1), FpMV1 (YP_009272898.1), FpMV2 (YP_009272899.1), FpMV3 (YP_009272900.1), FpMV4 (BAV56292.1), FsMV1 (QIQ28428.1), GaMRVS2 (YP_077184.1), GsMV1 (MN043682.1), HfMV1 (AIU44705.1), LbMV1 (YP_009553599.1), LjMV1 (MK279483.1), MpMV1 (ALD89100.1), MpMV3 (KT823703.1), NlMV1 (YP_009388498.1), NoMV1 (MH823901.1), NoMV2 (MH823902.1), NpMV1 (QDB74992.1), Oph.MV5 (NP_660180.1), OnuMV6 (NP_660181.1), Oph.MV1a (AM087548.1), Oph.MV3a (NP_660176.1), OsMV1 (MK279484.1), OsMV2 (MK279485.1), SnMV1 (ANJ77669.1), SnMV2 (ANJ77670.1), SsMV1 (YP_009121785.1), SsMV2 (YP_009551566.1), SsMV3 (CEZ26305.1), SsMV4 (AGC24233.1), SsMV5 (AHX84132.1), SsMV6 (AXI69836.1), SsMV7 (AHX84135.1), SsMV8 (AHF48624.1), SsMV9-A (AWY10972.1), SsMV11 (AHF48627.1), SsMV12 (AHF48628.1), SsMV14-A (AWY10977.1), SsMV15 (AHF48631.1), SsMV17 (ALD89134.1), SsMV18 (KP900925.1), SsMV19 (ALD89136.1), SsMV20 (ALD89137.1), SsMV27 (AWY10985.1), SsMV28 (MF444258.1), SsMV29 (AWY10985.1), SsMV30 (MF444260.1), StMV1 (AZT88625.1), TaMV (YP_004564622.1), and TbMV (YP_002822229.1).

### 2.5. Analysis of Fungal Vegetative Growth and Mycovirus Transmission to Conidia

**Figure 1:**
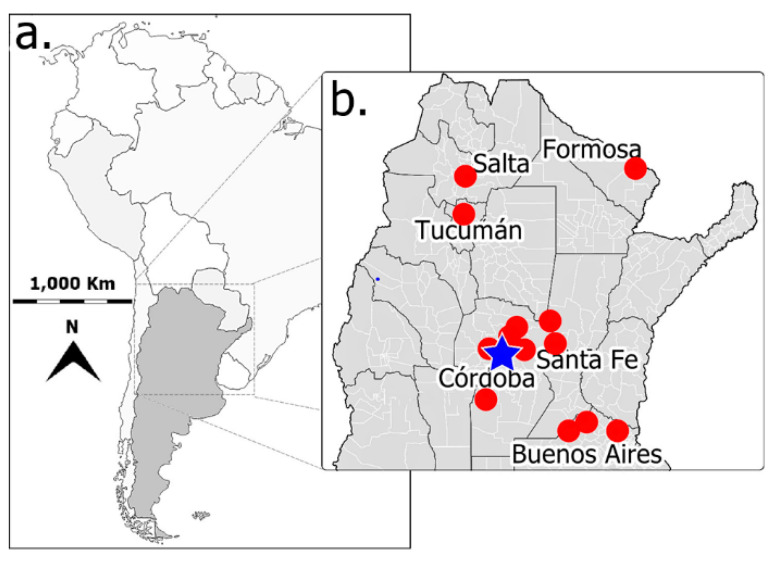
Source location of the *Fusarium* spp. isolates used for the mycovirus survey. (**a**) South America political map in which the Argentine national territory is painted in dark gray, and black lines indicate international divisions. (**b**) Zoom-in view of the central and northern regions of Argentina; red dots represent the areas where maize and sorghum grains were taken to sample for *Fusarium* spp. isolates. The blue star indicates the site where the *Fusarium* isolates infected with mycovirus were obtained (*F. verticillioides* isolate FvSec505 from maize and *F. andiyazi* isolate Fa162 from sorghum). Black lines indicate provincial limits, and white lines, departmental regions within provinces in Argentina (grey), whose names are indicated.

Growth rate and conidia production were analyzed in infected strains FvSec505 and Fa162, and the virus-free strains which were used for phenotypic comparisons. The virus-free strains selected from each *Fusarium* species were isolates confirmed to lack dsRNA bands consistent with viral infection. Regarding *F. verticillioides*, infected strain FvSec505, uninfected reference strain FvM3125, and a second virus-free strain, FvArv2300, collected from maize obtained in the same area that FvSec505 (Manfredi, Córdoba province, Figure 1), were included in the analysis. On the other hand, vegetative growth of *F. andiyazi* infected strain, Fa162, was compared with that of virus-free strain *F. andiyazi* 210 (Fa210), collected from sorghum obtained in the same area (Manfredi, Córdoba province, Figure 1). To assess growth rate, 10 µL conidia suspensions containing 1.0 × 10^4^ conidia/mL of each strain were inoculated into the center of PDA Petri dishes, which were then incubated in the dark for 7 days, at 25 °C. The radial growth was measured daily to determine the growth rate and lag phase. At the end of the experiment, plates were used to quantify conidia production. For that purpose, microconidia from each sample were harvested twice by adding 15.0 mL of Tween-20 (Sigma-Aldrich Corp., Burlington, MA, USA) 0.5% in sterile distilled water to the plate and rubbing the surface with a sterile bent glass rod. The obtained suspension was filtered through Miracloth (EMD Millipore Corp., Burlington, MA, USA) membranes, and the microconidia were counted using a hemocytometer [80]. Four replicas were performed for each strain, and the experiment was repeated twice. In the case of infected strains FvSec505 and Fa162, the conidia collected were also used to prepare monosporic subcultures to analyze mycovirus vertical transmission rates. A total of 50 monosporic cultures were performed for *F. verticillioides* strain Sec505, and 11 for *F. andiyazi* strain 162. Then, these monosporic isolates were analyzed by chromatography on cellulose, as described above, to determine the presence of the dsRNA band.

### 2.6. Fumonisins B Production

Fumonisins B (FBs) production was determined in *F. verticillioides* infected strain FvSec505 and virus-free strains FvM3125 and FvArv2300. To that effect, conidial suspensions (500 µL containing 0.5 × 10^6^ conidia/mL) of each strain were inoculated into 50 mL GYAM liquid medium (0.67 g malic acid, 1.2 g 1-asparagine, 0.0992 g NaCl, 0.766 g K_2_HPO_4_, 0.492 g MgSO_4_, 0.976 g CaCl_2_, 0.5 g yeast extract and 40 g glucose, per liter, adjusted to pH 3.0). Cultures were incubated in the dark, with shaking at 28 °C for 7 days. Then, 1000 µL of each liquid culture were centrifuged for 15 min at 9000 RCF. The obtained supernatants were diluted with HPLC grade acetonitrile (Sintorgan S.A. Villa Martelli, Buenos Aires, Arg.) at a 1:1 ratio, and the FBs content was determined in a Perkin Elmer HPLC equipped with a fluorescence detector, following the methodology proposed by Shephard et al. [81]. The quantification of FB_1_ was carried out by comparing the peak areas obtained from samples with FB_1_ analytical standards (PROMEC, Tygerberg, Republic of South Africa), using HP Chemstation Rev. A.07.01 software [82]. Five replicas were prepared for each sample, and the experiment was repeated twice.

### 2.7. Phytopathogenicity Assay

The virulence of infected and mycovirus-free strains towards maize seedlings (*Zea mays* L.) was assayed in a growth chamber under controlled conditions, according to the protocol by Arias et al. [82] with some modifications. Susceptible ACA474 Hib. seeds were harvested before the experiment and stored at −20 °C in a semi-permeable bag. Prior to the inoculation, seeds were superficially disinfected by immersion in 5.0% NaClO solution for 1 min, and rinsed three times with sterile distilled water. Seeds were inoculated by overnight incubation in a conidia suspension (1.0 × 10^6^ conidia/mL) of each strain. Ten seeds were used per treatment. Inoculated seeds were then placed in Petri dishes with moistened paper and incubated at 25 °C in darkness for 48 h. Then, the germinated seeds were transferred to hydroponic culture in a growth chamber with a photoperiod of 12 h of light and 12 h of darkness, relative humidity at 75%, and constant temperature at 25 °C. The irrigation and concentrations of the macro and micronutrients of the hydroponic solution were carried out according to Zörb et al. [83]. Two parameters were used to assay virulence, stem height, and plant biomass. Stem height data were recorded from each plant on day one, four, eight, and fifteen post-germination, considering day one when the coleoptile or the first leaf exceeded 5 cm in height. Measurements were taken from the seed to the distal end of the longest leaf. At the end of this period, the dry weight of the seedlings (biomass) was obtained after drying them at 60 °C for 7 days.

### 2.8. Statistical Analyses

Statistical analyses of fumonisin B1 production data and phytopathogenicity assay data were performed by one-way analysis of variance (ANOVA) (*p* ≤ 0.05). The normality and homogeneity of variance were tested. Data are presented as mean ± standard error, and differences between means were considered significant if probability *p* ≤ 0.05. DGC test was used for means comparisons. Statistical analyses of growth rate and conidia production were performed by one-way ANOVA (*p* ≤ 0.05), and Fisher test (*p* ≤ 0.05) was used for means comparisons. All statistical analyses were performed using InfoStat v2020 software (Córdoba, Córdoba, Arg.) [84].

## 3. Results and Discussion

**Figure 2:**
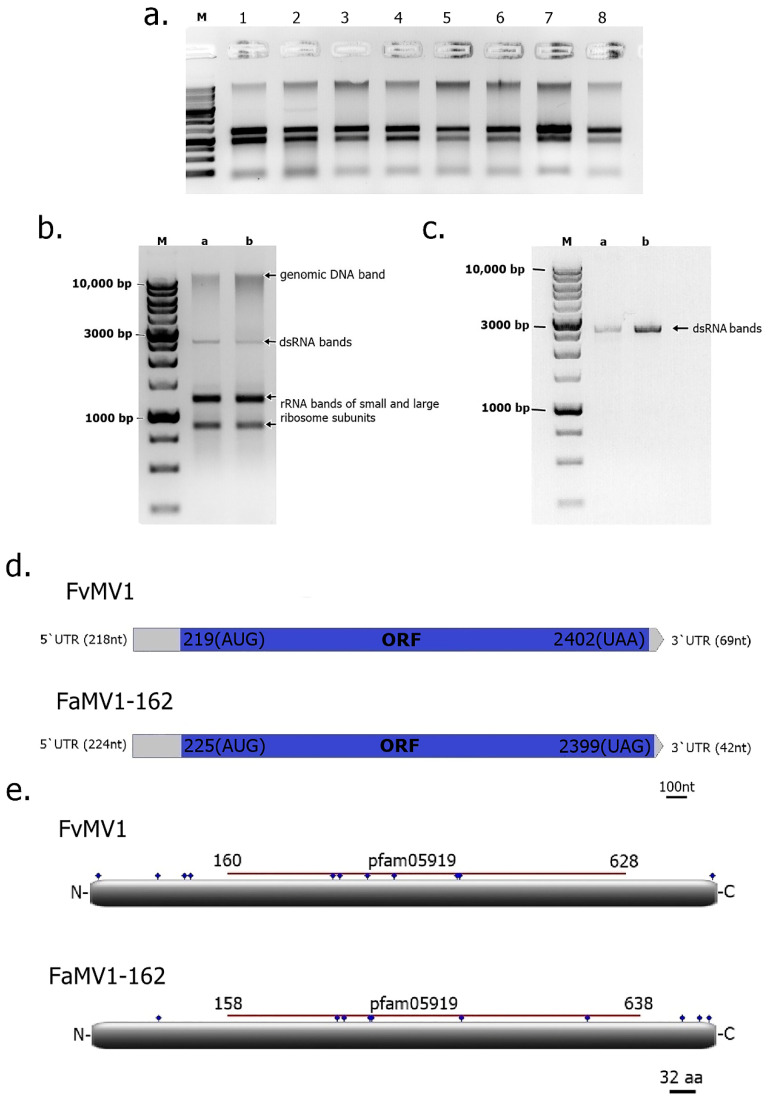
Molecular characterization of Fusarium verticillioides mitovirus 1 (FvMV1) and Fusarium andiyazi mitovirus 1 strain 162 (FaMV1-62). (**a**) Representative Agarose gel electrophoresis (0.8% *w*/*v* in buffer TAE) of chromatography on cellulose extracts prepared from monosporic cultures of fungal strains for the identification of dsRNA mycoviruses. Lane M: DNA molecular weight marker (1Kbp, NZYDNA Ladder III, NZYTech^®^ Paço do Lumiar, Lisboa, Portugal); lane 2: *F. verticillioides* isolate FvSec505; lanes 1 and 3 through 8: other *Fusarium* isolates from survey. (**b**) Agarose gel electrophoresis (0.8% *w*/*v* in buffer TAE) of chromatography on cellulose extracts from *Fusarium andiyazi* strain 162 (lane a) and *Fusarium verticillioides* strain Sec505 (lane b) showing dsRNA bands indicative of mycoviruses infection. (**c**) Agarose gel electrophoresis of the dsRNA bands identified in *F. andiyazi* strain 162 (lane a), and *F. verticillioides* strain Sec505 (lane b) after treatment with DNase and S1 Nuclease; as observed, both bands resisted digestion confirming their dsRNA nature. Lane M: DNA molecular weight marker (1Kbp, NZYDNA Ladder III, NZYTech^®^, Paço do Lumiar, Lisboa, Portugal). Gels were stained with ethidium bromide and nucleic acids visualized and photographed under UV light using a transilluminator (EC3 Imaging System—UVP, LLC). (**d**) Schematic representation of the genomes of FvMV1 and FaMV1-162. The blue rectangles represent the single ORF (5′ to 3′ sense) identified in each mycovirus. Start and Stop codons are noted in parentheses, and numbers represent their positions in the mycovirus sequenced genome. The grey rectangles represent the 5′- and 3′-untranslated regions (UTR) with their lengths indicated in parenthesis. Scale bar is drawn in nucleotide unit (nt). (**e**) Schematic representation of predicted RNA dependent RNA polymerase (RdRp) proteins encoded by FvMV1 and FaMV1-162. The gray box represents the full length predicted proteins from their amino-terminus (N-) to the carboxyl-terminus (-C). Blue dots indicate the positions of UGA-encoded tryptophan. The dark-red line drawn above the gray rectangles indicates the position (numbers indicate the aa position) of the conserved domain of the mitoviral RdRp Superfamily (pfam05919). Scale bar is drawn in amino acid unit (aa).

The advent and application of next generation sequencing techniques (e.g., RNA-seq and Metagenomic) have greatly increased the pace of mycovirus discovery in a wide diversity of fungi [5,6]. Up to date, mycoviruses had been identified in 16 different *Fusarium* species [52,58,77,85] including *F. andiyazi*, *F. asiaticum*, *F. boothii*, *F. circinatum*, *F. coeruleum*, *F. globosum*, *F. graminearum*, *F. incarnatum*, *F. langsethiae*, *F. oxysporum*, *F. poae*, *F. pseudograminearum*, *F. sacchari*, *F. solani*, *F. culmorum* and *F. virguliforme*. However, no mycovirus had been reported so far in *F. verticillioides*, the major causal agent of corn ear rot and fumonisin producer in the grains. The primary goal of this study was to identify and characterize mycoviruses in the fungal pathogen *F. verticillioides*. Consequently, we generated a large collection of *Fusarium* isolates sampled from maize kernels, both with and without disease symptoms, collected in different areas of Argentina (Figure 1). Isolates with the macroscopic appearance of *F. verticillioides*, which is the predominant *Fusarium* species in maize from the temperate region [86,87], were selected, and analyzed by PCR using specific primers for *F. verticillioides* (see Materials and Methods for details). Moreover, six isolates morphologically identified as *F. andiyazi*, an important *Fusarium* pathogen in sorghum [88,89], were also included in this survey. The identification of dsRNAs in fungal strains generally indicates mycovirus infection, as fungi lack large endogenous dsRNA molecules (larger than 100 nt) [90,91]. To test for the presence of dsRNAs in our fungal collection, chromatography on cellulose extracts were prepared for each isolate and analyzed by agarose gel electrophoresis (Figure 2a). Large dsRNA molecules compatible with viral genomes [92] were identified in only 2 out of 105 *Fusarium* isolates tested (99 *F. verticillioides* and 6 *F. andiyazi*) (Figure 2b). These were *F. verticillioides* strain Sec505 (FvSec505) and *F. andiyazi* strain 162 (Fa162), obtained from maize and sorghum, respectively, in Manfredi, Córdoba province (Figure 1) (see abbreviations in Table 1). In both strains, FvSec505 and Fa162, a single dsRNA molecule of approximately 2.5 kilobase pairs (kb) was detected (Figure 2b). Treatment of samples with DNase I and Nuclease S1 did not degrade the observed bands, confirming their dsRNA nature (Figure 2c). Furthermore, these dsRNAs bands were stable through repeated subculturing of the host fungal strains.

**Figure 3:**
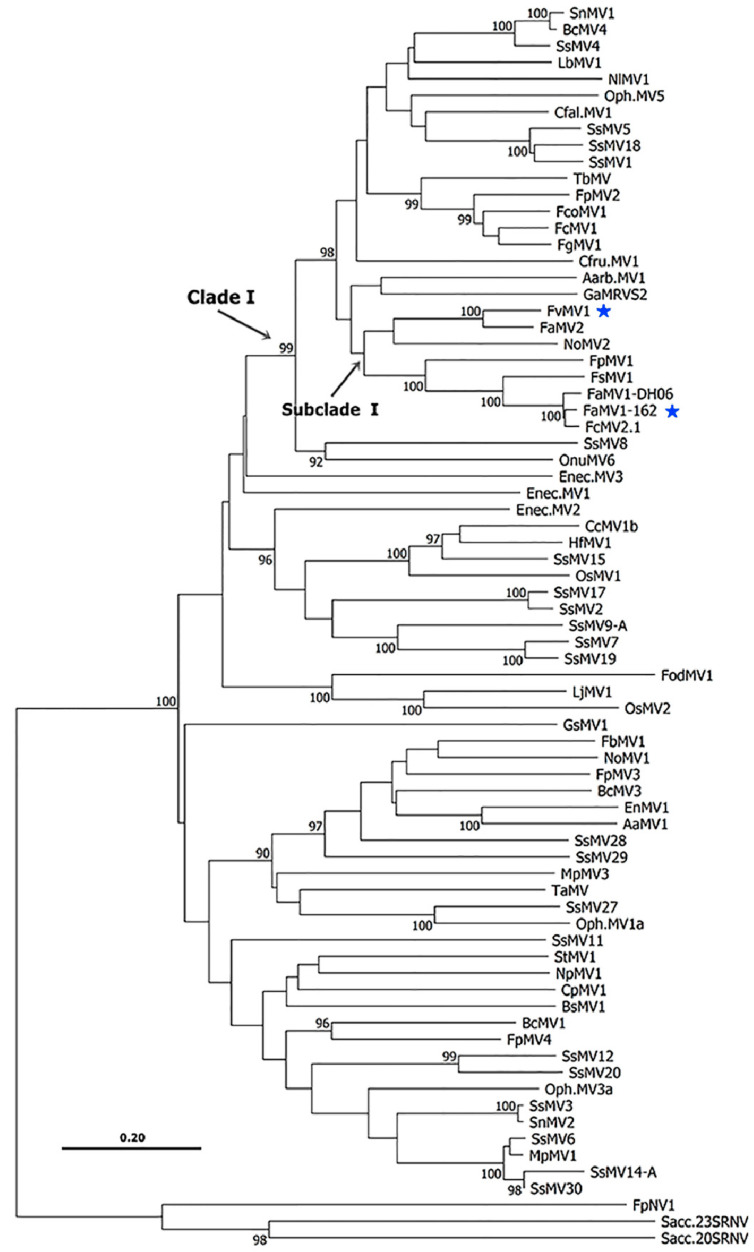
Phylogenetic analysis of mitoviruses (*Mitoviridae*) isolated from fungi of the Ascomycota phylum. The tree is displayed as a rectangular phylogram of 73 mitoviruses and rooted on the branch to members of the genus *Narnavirus* (Narnaviridae) as external group (FpNV1: YP_009272902.1, Sacc.20RNV: NP_660178.1 and Sacc.23RNV: NP_660177.1). The tree was inferred using the Neighbor-Joining method, based on multiple sequence alignments through MAFTT software (L-INS-i, automatized election). The evolutionary distances were computed using the Poisson correction method (uniform rates between sites), and are in the units of the number of amino acid substitutions per site. All positions containing gaps and missing data were eliminated (complete deletion option). The percentage of replicate trees in which the associated taxa clustered together in the bootstrap test (1000 replicates) is shown as node labels (values are in percentage and less than 90% were hidden in the graph). The scale bar represents substitutions per site. The viruses identified and characterized in this study are indicated with a blue star.

To characterize dsRNAs at the molecular level, we performed deep sequencing of total RNAs depleted of rRNA in the strains harboring them, FvSec505 and Fa162. Illumina NovaSeq HTS of *F. verticillioides* strain Sec505 RNA, and sequence analysis (see Materials and Methods) identified a single virus-like contig 2471 nt long, with a 28.7% GC richness. This putative new mycovirus was found to contain a single ORF (genetic code 4) 2184 nt in length, expanding from nt positions 219 (AUG) to 2402 (UAA), flanked by 5′- and 3′-untranslated regions (UTRs) 218 nt and 69 nt in length, respectively (Figure 2d). The genome size of 2471 nt determined by NGS is in line with the one predicted by the aforementioned electrophoretic analysis (Figure 2c). Given the high support of virus reads, the presence of typical UTR sizes, and a total length consistent with that of other similar viruses [93], we entertain the hypothesis that the determined virus sequence is coding complete (CC) and nearly complete. A similarity search using BLASTp against the nr NCBI database showed that the predicted aa sequence of the protein encoded by the single ORF shared significant sequence identity with the RdRps of viruses in the genus *Mitovirus*, family *Narnaviridae* (recently changed to *Mitoviridae*—*Cryppavirales*). Moreover, a conserved domain search (CDD, NCBI) indicated that it contained a conserved motif of the Mitovirus RNA dependent RNA polymerase Superfamily (Accession: cl05469; E-value = 1.13 × 10^−113^) (Figure 2e). This deduced protein sequence has 727 aa with a molecular mass of 84.85 kDa. The virus identified in the *F. verticillioides* strain FvSec505 exhibited the highest similarity to Fusarium andiyazi mitovirus 2 strain FS09 with 83.63% identity. The species demarcation criteria in the genus *Mitovirus*, defined by the ICTV 9th Report (https://talk.ictvonline.org/), indicate that mitoviruses with homologies in amino acid sequence of RdRp proteins greater than 90%, belong to different strains of the same mitovirus species. Therefore, we can conclude that the dsRNA segment identified in the *F. verticillioides* strain FvSec505 corresponds to a novel mycovirus, a tentative member of a new species, belonging to the genus *Mitovirus* of mitochondrial mycoviruses with +ssRNA genomes. We have named this novel mycovirus, the first reported in this fungal pathogen, Fusarium verticillioides mitovirus 1 (FvMV1). A phylogenetic analysis (Figure 3) clustered the novel mitovirus FvMV1 isolated from *F. verticillioides* (Subcl. Hypocreomycetidae) with mitovirus FaMV2 isolated from *F. andiyazi*, and with the mitovirus NoMV2, isolated from a species of other fungal genus, *Nigrospora oryzae* (Subcl. Xylariomycetidae). Sequence identity between FvMV1 and NoMV2 was, however, clearly lower than that between FvMV1 and FaMV2, 50.28% and 83.36%, respectively.

In the *F. andiyazi* strain Fa162, HTS of RNAs and sequence analysis also identified a single virus genome, 2441 bp in length with high AT richness (GC = 29.5%). The mycovirus genome was found to contain a single ORF (genetic code 4) of 2175 nt, extending from nt 225 (AUG) to nt 2399 (UAG) (Figure 2d), flanked by 5′- and 3′-UTRs 224 nt and 42 nt in length, respectively. This single ORF encoded a 724 aa protein, with a molecular mass of 84.63 kDa. A similarity search showed that this protein shared strong similarity with the RdRps of mitoviruses. A conserved domain search (CDD, NCBI) also identified a motif conserved in the Mitovirus RNA dependent RNA polymerase Superfamily (Accession: cl05469; E-value = 9.64 × 10^−118^). The predicted protein encoded by this mycovirus was found to be most similar (95.44% identity) to the RdRp of Fusarium andiyazi mitovirus 1 (FaMV1) (accession: QIQ28423.1). Therefore, according to ICTV rules, the mycovirus identified in *F. andiyazi* in this work represents a new strain of a previously identified mitovirus, which we have named Fusarium andiyazi mitovirus 1 strain 162 (FaMV1-162). FaMV1 was identified in a recent study on sugarcane pathogens in China, by Yao and coworkers [77], together with a second mitovirus, Fusarium andiyazi mitovirus 2 (FaMV2). Interestingly, FaMV1 and FaMV1-162 also shared a 95.44% identity with a mitovirus identified in a different *Fusarium* species, Fusarium circinatum mitovirus 2-1 (FcMV2.1) isolated from the conifer pathogen *F. circinatum* in Spain [94]. Considering the high aa sequence identity, these mycoviruses isolated from two different *Fusarium* species represent three strains of the same mitovirus species. In some fungal genera, like *Heterobasidium*, mycoviruses appear to be readily transmitted between species [95]. Future studies should be carried out in order to evaluate the mycovirus interspecific transmission between these *Fusarium* species with close phylogenic relationship.

Members of the genus Mitovirus are characterized by having a mono-segmented and non-encapsidated +ssRNA genome, so-called naked RNA replicons, and a subcellular localization in the host mitochondria [93,96]. Their genome is generally small in size, ranging from 2.0 to 4.5 kb, and rich in A-U (generally > 60%), with a single long open reading frame (ORF) that encodes an RdRp [96]. The genomes of FvMV1 and FaMV1-162 have these typical features of mitoviruses with sizes close to 2.5 kb, and 71.4% and 70.6% AU content, respectively. The UTRs of mitoviruses usually form looped structures with dsRNA regions, which are proposed to be involved in replication [93]. We predicted that the 5′- and 3′-UTRs of FvMV1 and FaMV1-162 have the ability to self-fold to form looped structures with dsRNA regions stable at room temperature (Supplementary Figure S1). Another feature of mitoviruses is that they contain a high amount of UGA codons in their genome [96]. The UGA codon is a stop signal in the mRNA translation system of cytoplasmic ribosomes. However, in the mitochondria of filamentous fungi, UGA is the codon for the amino acid tryptophan [97]. The replacement of UGG by UGA as the codon for tryptophan in the genome of the member of the genus Mitovirus, as a consequence of the mitochondrial translation system selective pressure [98], implies that they cannot replicate in the cytoplasm. In accordance with the mitovirus replicative cycle, two artificial groupings could be established. One includes mitoviruses that can reproduce theoretically in both the mitochondria and cytoplasm (ORF with relation UGA/UGG = 0), while a much broader one includes mitoviruses that can be only reproduced in the host mitochondria (ORF with UGA/UGG ratio > 0). In the genome of FvMV1, 100% of the tryptophan (11/11) is encoded by a UGA codon. Similarly, in the FaMV1-162 genome, 92% of the tryptophan (11/12) is encoded by a UGA codon. These results indicate that FaMV1-162 and FvMV1 might replicate exclusively in the fungal host’s mitochondria. Furthermore, this highly probable exclusive mitochondrial localization of FaMV1-162 and FvMV1 could explain their very efficient transmission to conidia, since during formation of conidia, it necessarily receives one or more mitochondria from the conidiophore hypha and, therefore, the legacy of the Mitoviruses.

**Figure 4:**
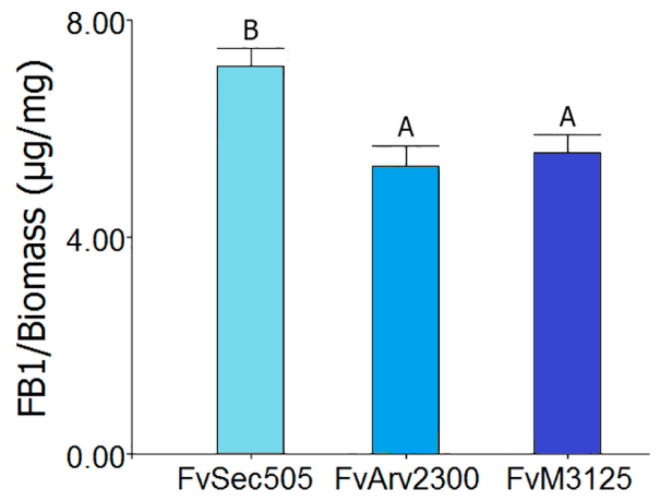
Fumonisin B1 (FB_1_) production of *F. verticillioides* strain FvSec505 infected with FvMV1 compared with two virus-free strains, FvM3125 and Fvarv2300. FB_1_ production (µg/mg of mycelium biomass) in GYAM culture medium at 25 °C was expressed as means and SE. Bars with different letters are statistically different from each other, according to the DGC multiple range test (*p* ≤ 0.05). Whiskers show standard error. Five replicas were prepared for each sample, and the experiment was repeated twice.

**Figure 5:**
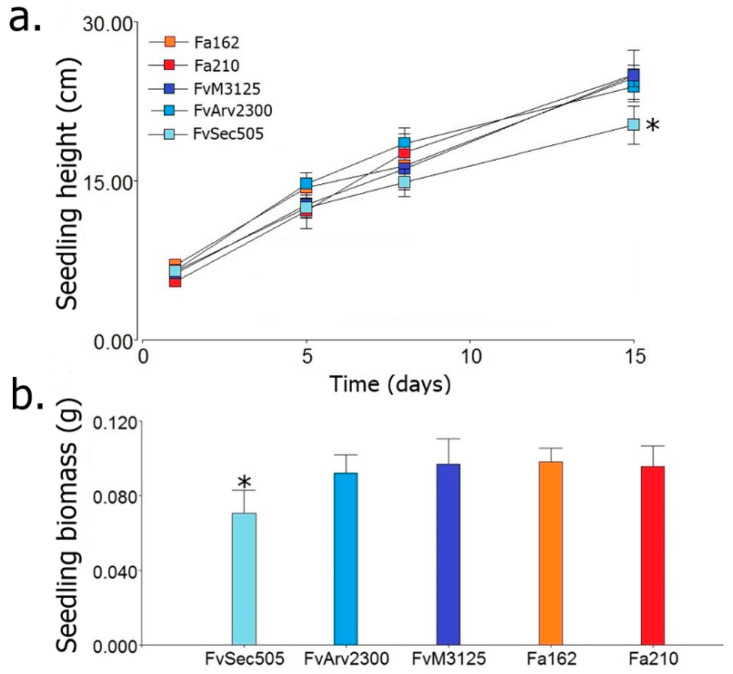
Phytopathogenicity of infected and mycovirus-free strains towards seedling of *Zea mays* L. (**a**) Growth of corn seedlings infected with the different fungal strains. The inoculation was carried out in the seeds, and the seedlings were cultivated in a hydroponic system with controlled photoperiod, temperature, and humidity. The height of the plant stem was measured after germination, when the coleoptile or the first leaf exceeded 5 cm in height. Measurements were taken on day one, four, eight, and fifteen post germination. The values are expressed as means of plant height, and the whiskers show standard error. The asterisk indicates a significant difference with respect to the other measurements on the same day (multiple range DGC test, *p* < 0.05). (**b**) Plant biomass of infected seedlings. Values are expressed as biomass mean, and whiskers show standard error. The values containing an asterisk are statistically different from the others, according to the multiple range DGC test (*p* < 0.05). In panels (**a**,**b**), *Fusarium verticillioides* and *F. andiyazi* strains were evaluated separately. The mycovirus-infected strains are *Fusarium andiyazi* 162 (Fa162 with FaMV1-162) and *F. verticillioides* Sec505 (FvSec505 with FvMV1), and the virus-free strains are *F. andiyazi* 210 (Fa210), *F. verticillioides* M3125 (FvM3125) and *F. verticillioides* Arv2300 (FvArv2300).

**Figure 6:**
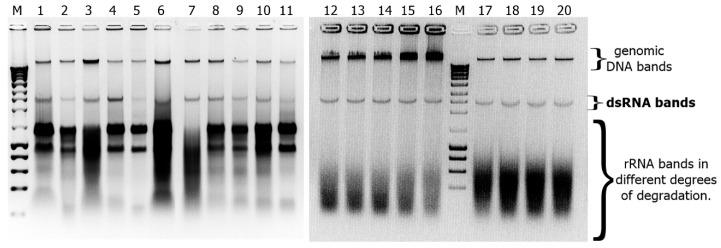
Agarose gel electrophoresis (0.8% *w*/*v* in buffer TAE) of chromatography on cellulose extracts prepared from monosporic cultures of mycovirus-infected fungal strains. Lanes 1 through 11: extracts from monosporic cultures prepared from *F. andiyazi* strain Fa162; lanes 12 through 20: extracts from monosporic cultures prepared from *F. verticillioides* strain Fv505; Lanes M, 1 Kb DNA molecular weight marker (NZYDNA Ladder III, NZYTech^®^, Paço do Lumiar, Lisboa, Portugal).

Mitoviruses are widely distributed in nature [76,99,100], including a great diversity of fungal species [96]. Mitoviruses are the most common mycoviruses isolated from *Fusarium* species [52,77,96]. Infections caused by the majority of the mitoviruses reported so far are asymptomatic. However, some mitoviruses that produce phenotypic alterations have been identified. Wu et al., [35,101] reported hypovirulence and growth alterations caused by the mitovirus BcMV1 in *Botrytis cinerea*. A similar effect was attributed to SsMV1 infection in *Sclerotinia sclerotium* [102]. Alterations in the ultrastructure, size, and quantity of mitochondria have been associated with the presence of mitoviruses TbMV [103] and BcMV1 [101] in their natural hosts *Chalara elegans* (Thielaviopsis basicola) and *Botrytis cinerea*, respectively. As a first approach to determining if the identified mycoviruses might induce alterations in the host, we compared the phenotype of FvSec505 and Fa162 with that of mycovirus-free isolates of the same species. The phenotypic characterization included analysis of growth rate, lag phase, conidia production (Table 2), FB_1_ production (Figure 4), and pathogenicity (Figure 5). In *F. andiyazi*, FaMV1-162 infected strain Fa162 and a virus-free strain sample in the same area, Fa210, exhibited no significant differences in growth, conidiation, or virulence. These results are in agreement with those by Yao et al., [77] who reported that FaMV1 caused asymptomatic infection in *F. andiyazi*. In contrast, the *F. verticillioides* strain Sec505 infected with the novel mitovirus FvMV1 showed clear phenotypic differences with two virus-free *F. verticillioides* strains, FvM3125 and FvArv2300. Interestingly, what we observed was a significant increase in conidia and FB_1_ production in the *F. verticillioides* strain FvSec505 harboring mitovirus FvMV1, compared with the two uninfected strains (Table 2 and Figure 4). We also observed an increase in virulence in the presence of FvMV1. Thus, there was a significant reduction in the seedlings growth rate and seedling biomass when seeds were inoculated with FvSec505 conidia, compared with FvM3125 and FvArv2300 infections (Figure 5a,b). Several studies have shown that fumonisin production is a key factor involved in the *F. verticillioides* pathogenicity [104,105]. Hence, the relatively higher virulence of FvSec505 and its increased FB_1_ production could be linked. Observation of phenotypic alterations in an infected strain can be an indication that this virus infection has effects on the host. However, to confirm that those phenotypic alterations are caused by the mycovirus, isogenic infected and virus-free strains have to be generated. To our knowledge, there are no reports on mitoviruses being linked to increased severity of fungal disease. The molecular analysis of mycovirus–fungus interactions that result in increased virulence and fumonisin production can provide new insights into the regulation of these processes. As a result, we considered of interest to conduct the analysis of the FvMV1–*F. verticillioides* interaction. Bearing this in mind, single spore cultures of FvSec505 were produced as an avenue to select a virus-free version of this strain, and also to analyze the transmission rates of FvMV1 to the next generations (vertical transmission). Single spore cultures of *F. andiyazi* Fa162 infected with FaMV1-162 were also produced to get a general picture of vertical transmission rates to the conidia of both mitoviruses identified in this study. Analysis of monosporic cultures by chromatography on cellulose showed that all of them still contained the virus (Figure 6). Therefore, unfortunately, production of monosporic cultures of FvSec505 and Fa162 failed to generate a virus-free version of these strains, since the transmission rate of FvMV1 and FaMV1-162 to conidia was 100%. In addition to single spore cultures, a hyphal tip isolation strategy was also developed in order to obtain infected and uninfected isogenic strains. However, this was not successful. These results led us to conclude that FvMV1 infection is very stable in the fungus tissues. Other approaches to generate strains for the analysis of *F. verticillioides* phenotypic traits in the presence/absence of FvMV1 in the same genetic background are being implemented.

The present survey of mycoviruses in *F. verticillioides* isolated from maize produced in Argentina showed a very low incidence of virus infection in this fungal species; only 1 out of 99 isolates (1.01%) analyzed by chromatography on cellulose was confirmed to harbor a mycovirus. This is a quite low viral infection rate compared with that found in other *Fusarium* species, although it should be noted that numbers greatly varied in different studies. For instance, a survey in China using RNA-seq technology identified mycovirus sequences in 9 out of 41 *F. andiyazi* isolates (21.9%) and 6 out of 42 *F. sacchari* isolates (15.3%) analyzed [77]. Surveys in Korea and Iran have shown 2.3% and 3.6% incidence of viral infection in *F. graminearum* isolates from maize, respectively [58]. In contrast, incidence of viral infection was found to be very high in a collection of *F. virguliforme* isolates, where 23 out of 44 isolates (52%) showed presence of mycoviruses [106]. On the other hand, in *F. andiyazi,* we found that 1 out of 6 isolates from sorghum analyzed was infected. However, we consider that the sample was too small and not representative enough to allow us to draw any conclusion regarding the mycovirus infection rate of this *Fusarium* species infecting sorghum in Argentina. Hyphal anastomosis is the main route of mycovirus horizontal transmission [3,20]. This process is regulated by a series of fungal loci, designated hsi (heterokaryon self-incompatibility), het (heterokaryon incompatibility), vic (vegetative incompatibility), or sup (suppressors) [107]. *Fusarium verticillioides* has at least 10 vic loci, and assuming that each vic locus in the population segregates only two alleles, the number of VCGs that theoretically can be found in the population is 2 × 10^10^ = 1024 [72,107]. A study carried out by Chulze et al. [108] in Córdoba, Argentina, on maize sampled within a 50Km diameter area, indicated that in 36 *F. verticillioides* isolates tested, the VCG/isolate ratio was 0.77, that is, it was rare that two or more isolates belonged to the same VCG [108]. It can be hypothesized that the highly complex VCG structure contributed to limit the spread of mycoviruses in this fungal pathogen. However, it is unlikely that a complex VCG structure alone can explain the low incidence of viral infection we reported here for *F. verticillioides*, since there is little evidence in the literature of a clear correlation between mycovirus incidence and the complexity of VCG structure [109,110]. Additional mycovirus surveys will be required to determine if the low incidence of viral infection in *F. verticillioides* reported here extends to other world regions. We will conduct future studies on the transmission of FvMV1 and FaM1-162 to the ascospores, since sexual reproduction in *F. verticillioides* is known to be relatively frequent in the fields of central Argentina [108], and sexual reproduction might provide an avenue for mycovirus transmission between vegetative incompatible fungal strains [111].

In the coming years, a higher incidence of *Fusarium* diseases is expected in most corn-producing regions, due to a prevailing trend towards higher temperatures, higher evapotranspiration, and an increase in the frequency of extreme weather events [112,113]. Due to the low efficiency of fungicides in reducing *F. verticillioides* disease symptoms and fumonisin contamination, and their negative environmental impact, there is a need to develop new disease management approaches. The use of mycoviruses represents an interesting biological control strategy to explore. The development of efficient mycovirus-based control strategies requires the analysis of important aspects, such as efficiency of mycovirus transmission and the mechanisms that control the mycovirus–host interaction leading to stable virus infection in fungal cells. Furthermore, mycoviruses that induce alteration in growth, virulence, and/or mycotoxin production might provide an interesting tool to shed light on the molecular mechanisms that control these processes. To our knowledge, the results presented here represent the first contribution to the study of mycoviruses in the major fungal pathogen *F. verticillioides*. The findings reported include the analysis of the mycovirus infection rate in a large collection of isolates of *F. verticillioides*, the molecular description of the first mycovirus isolated from this species, the study of its transmission rate to conidia, and the uncovering of potential phenotypic effects on the host, which are worth further exploring. A strategy based on protoplast fusion is being developed in order to obtain the infected and uninfected *F. verticillioides* isogenic strains (same genetic background), and to evaluate the effect of FvMV1 on fungal growth, fumonisin production and fungal pathogenicity in maize. Furthermore, future study on the transmission of FvMV1 to the sexual spores of *F. verticillioides* will be carried out to better understand the biology of the fungus–virus interaction.

## Figures and Tables

**Table 1:** Abbreviations.

Abbreviations	Meaning
FB	B-series fumonisins
(+)ssRNA	positive sense single-stranded RNA
dsRNA	double-stranded RNA
FvSec505	*F. verticillioides* strain Sec505 (infected with FvMV1)
FvM3125	Virus-free strain *F. verticillioides* M3125
FvArv2300	Virus-free strain *F. verticillioides* Arv2300
Fa162	*F. andiyazi* strain 162 (infected with FaMV1-162)
Fa210	Virus-free strain *F. andiyazi* 210
FvMV1	Fusarium verticillioides mitovirus 1
FaMV1-162	F. andiyazi mitovirus 1 strain 162

**Table 2:** Growth rate, lag phase, and conidia production of the mycovirus-infected and mycovirus-free strains.

*Fusarium* Isolate	Growth Rate (mm/Day)		Lag Phase (Hour)		Conidia/mL/mm^2^
	PDA	CDA	PDA	CDA	
**FvM3125**	4.89 ± 0.09a	6.06 ± 0.10a	46.58 ± 1.13b	31.58 ± 0.85a	3303.85 ± 463.13a
**FvSec505**	6.53 ± 0.09b	7.09 ± 0.0c	35.40 ± 1.04a	31.38 ± 0.95a	5346.24 ± 463.14b
**FvArv2300**	6.97 ± 0.08c	6.65 ± 0.10b	37.73 ± 0.90a	28.88 ± 0.95a	3797.20 ± 463.14a
**Fa162**	6.20 ± 0.08y	8.18 ± 0.09z	32.88 ± 0.96z	31.35 ± 0.62z	4472.34 ± 401.94z
**Fa210**	6.79 ± 0.08z	7.15 ± 0.10y	32.31 ± 0.90z	31.22 ± 0.60z	5006.96 ± 401.94z

Growth rate, lag phase, and conidia production of the mycovirus-infected strains (*Fusarium andiyazi* 162, Fa162, with FaMV1-162 and *F. verticillioides* Sec505, FvSec505, with FvMV1), and of the virus-free strains (*F. andiyazi* 210, Fa210, *F. verticillioides* M3125, FvM3125, and *F. verticillioides* Arv2300, FvArv2300). Values were expressed as means ± standard error. The infected FvSec505 strain was compared with the virus-free FvM3125 and FvArv2300 strains. In addition, Fa162 was compared with the virus-free Fa210 strain. Values having different letters are significantly different between treatments, according to the Fisher test of multiple ranges (*p* ≤ 0.05). The experiments were performed twice with 4 replicates for each strain.

## Supplementary Materials

The following are available online at https://www.mdpi.com/1999-4915/12/10/1161/s1. Figure S1: Predicted secondary structures (2-D representation of this self-folding) of the terminal untranslated regions (UTR) of FvMV1 and FaMV1-162.
