# Supplementary material for: A Survey of Mycoviral Infection in Fusarium spp. Isolated from Maize and Sorghum in Argentina Identifies the First Mycovirus from Fusarium verticillioides
Source: Viruses. 2020 Oct 14;12(10):1161. doi: 10.3390/v12101161 (PMC7602464; doi:10.3390/v12101161)
Supplement: Supplementary file 1 [file viruses-12-01161-s001.pdf]

viruses

MDPI

Article

A Survey of Mycoviral Infection in *Fusarium spp.* isolated From Maize and Sorghum in Argentina Identifies the First Mycovirus from *Fusarium verticillioides*

Andrés G. Jacquat, Martín G. Theumer María C. Cañizares, Humberto J. Debat, Juliana Iglesias and María D. García Pedrajas, José S. Dambolena.

1. Supplementary Data

1.1. Complete genomic sequence of Fusarium verticillioides mitovirus 1 (NCBI-GenBank accession numbers MT506024):

>FvMV1

CCATTAGTTAATAATAATCTTGGGAATTTAGTCATTTGACTAAATTGTCCCATAGGACTAAT
GATAATTTCTGTATTATCATTACCATTAAAGAATCGGTTAGAACCCGTATCTCTATGGTTTTA
TCCTGTCTGAGTGGTTAGTCAGAATCTGACTAATTTAATCAGGTTGCAGGATAGGGCTTATA
CACCCTAAAGTATAGTTTTAATATACCAATATGAAGAAAAATTATACCAAAATTCTTTGAA
GAATAATGGTTTTAATCTTTCCGTCTATTGATACATCAAAATACCTCCGTCCATTATTCAAA
ATCATCTTTAAAATGATAAAGAATCATGGAACTATTTATACCATCAAGTATTTAAAAAGAG
TAAGACTCCACTGTACAAGGTACATTTGTGGAGATCCTCTTTTTACAAATACTATGATGATA
GGAATAGATAAGGAGGGTTGACCAAAAGTTTTCTCATTCCTTAAACCATTAGTTAATAATA
ATCTTGAATCATTAAAATTCCTTTTTACCATTCTTAACTTCACAAGAAGTTGAGATTTGTCTA
AAAAGGAATGAGACAAGATAAAACCTGATTATAATAGTATAACAGACAAATCTAAAATGT
CTATTACTATTCCTTCAGGTATTATTAACAAATTTGTTAAGGAATACAGGTTAAAGTCTAAC
CATCCTGAATTTGATAAATTGAAAGATGTTTATCTTTCAACAAAAGCAGGACCAAATGGTC
CAGCTACTTTATCATCTCAGGAAGATCTGTTAAATTTTAATTATCCAATGATGGATAAAATA
TTTAAAATTACAGATCAAAATGGTATAGATTTCTTCTGTAAAAATTATACTCAAGCATTTAA
CAAAAATATTACTCCAAGTAAAATTAGAACACTTGGAAAAATATCTTTTGTTAAGGACCCT
GAATGTAAATTAAGAATAATTGCTATATCTGATTATTATAGCCAATTATATCTTAAACCTAT
ACATAATATTATCATGAATAAACTTCATAATATTACTATGGATAGAACTTATACTCAGGATC
CTCATCATGTTTGAGAAATAAATAATGAGAAATTTTGATCTCTAGACCTTAGTTCAGCAACA
GATAGATTTCCTGTAGAATTACAGAAAAGACTTTTAGCTAGAATATTCCATATGGAATTAGC
TCAAAGTTGACAATCTATTCTGAATTCAAGAGAGTTCACGACTCCAGAAGGTTACCAGTTA
AAATATGCAACTGGACAACCCATGGGAACGTATTCCTCTTGAAGTGTTTTCACCTTGACTCA
CCATTTAGTTGTATATTATTGTGCAACTATAAATGGCTACAAGAACTTTGACCAATATATAA
TTCTTGGTGACGATATCGTTATAAAAAATGATAAAGTTGCTAAGACTTATAAAAAGGTCATT
AAAGCTCTTGGAGTCGAATTATCTGAAAGTAAAACACATGTATCATCAAATACATATGAAT
TTGCTAAAAGATGAATTCAATGAAGTCAAAACCGTGAGATAACTGGACTTCCTCTTGGAGG
TATTCTTCGTAATATAAATAATCCTAATATAGTTTTCACTGTATTATACGATTATTTTAAAAT
TAAGAAGAATTATTTTCCAAGTAGGGCCAATTCTTTAGTAGGATTGGTTAAGGATTTATACC

ATAAATTATTATTAAAAAATAAAAAGATTAAAACTAAAAATAATAAAATTATTTTTAGAAA
ATCTTTTTATAAGCTTAATGATAAAATGGTATCATCTCTAGAGTCATTCTCCTTGATGTTAGA
TATAGTCTTTGATTATAAGTCTTACGATAAGTTAAGAAACTTATTCGTTAAGAATATTACAA
CCTTAGACTATAATATTCCAAGTGAAAGTACAATCCTTTCCGAATTGAAAAGGATACTTTCT
CATGGACTATCATCAAGAATTTTACAGATGAATAGTAATATAATAAATTCACCTAAAGTCC
TAATATCTAAATTTGATATTGAGGACAAGAATTTATTAAATACTAACCCTGTGTTCCTTTCC
ATCTATAACACAATCTCCCGATTTAAAGATATTAAATTTGAAGATCTAGACGATCTTCATAA
TATCTCTAAGGAGATATGTGAAATCAATATTGAAAATATTTTTAATAAGGATAGGAACAAG
ATCCAATCATTATTAGAAATAGGTAAAATATTGAAAGATGGGTTTAAATATGTCAACAATA
CAACTGAAGTGTATTATGGGTCAGCTACATTAAGCGACTCATTTACACTTGGTGGTATTGGT
AAAATTCTGATGAATAATTTAAAATCATCAGAATTAGACGACATAATTAAAGGAACTTACA
CTGAGCCACAGCCACAAGGTGGTTATGCTTCAATGTGAGAAAACTTTAAGATGTAATCCCT
AGACAATGATTGATTAAAATCATTATCTCCACTAACCATAGTGGGGAATCTAGTCAGAAAT
GAC

1.2. Complete genomic sequence of Fusarium andiyazi mitovirus 1 strain 162 (NCBI-GenBank accession numbers MT506025):

>FaMV1-162

TATCAATTAAGATAGACTCAATAGGTCCAAAGACCATTATACAGTTTAATTTCTGTATAATC
TAATGAAAGAAAGGTATAGCAAATCCTTTCTCTTTGGTCTTCCAAGAGTCCATATAATAATG
GGTTGCTCTTGGATAGCCGAGTGGCTTCTCGTTAAACAGTTAGACGGTAGGACTGCGAGTTA
AATACTCGAGTGAACATCCGCAACATTCAAATTTTATAATGTTAAGAAATTATATAAAAAT
AATCAAAAGATTAAGTTTTATATTTTTCCCTAACAAATACCATAAGGATGATTTTAATAAAA
TGCTGAAATTATATCAACATCTTATTAAACATCATAATATATCCGGTGCAATTAAATATATG
AAAAACATACGTTTAATTTGTACAAGATATATTTGTGGTAATCCATTATTAACTAATAATTTT
GGAATTGCCACAACCAATGGTTGACCAAATAAACTCTCATTCTTGAAGTCTAGGATTGATA
GTAATGAAGGTTTATCATACGTTCTTACTCTTCTAATATTTAATAGATCATTAGATCTAAATA
AATACGAAGTTAAGAAGAAAATGAAAAATCTTGATTATTCATCAATTACTTCTAAACAAAG
AATGAATTATACAATACCAACCGGTTTTATCAAAGAATTTGTAAAGAAGAATAATCTTTAC
ATTTCCAAAGATAAAATGGAGTTTTCCCTGAGTGACATTTATTTATCACAAAAAGGAGGAC
CACAAGGAAAAGCATCAAATACGGCTTTAGTAAATTTTAATAATTATAATTATTATAGTTTA
CAAAGACTATTTAATGTCCTTAGTGAGTCCGGTATTGATTTCATTTGTAAATCCTATAGTTAT
TTTGTGGAAAATTCTTCAAAATTTAAACCTAAACATAATGATTTAGGAAAGATTGAAGTTGT
AAAAGATCCAGAGGGTAAATTCAGATTAATTGCAATAGTTGATTACTATACACAATTAGCT
CTAAAGAAACTCCATGATCAGTGTTTTAAGGTTATAAAAAATCTTAAAGAAACTGATAGGA
CTTTTACACAAAATCCACATCATAACTGAGAATCTAATCAACATAAGTTTTGATCATTAGAT
TTGAGTTCTGCTACGGATAGATTCCCAAGGAAACTACAAGCAAGATTACTATCAGAAATGA
TAGATATGCATTATGCATGATCTTGAAATAGAATCCTTGAGACAATAAGTTTCCATACAAA
GGATGGCAACTCCATTAGATATGAAGTTGGCCAACCTATGGGAACTTATTCCTCCTGAATTT
GTTTCACTTTAGCTCACCACCTAGTAGTTAACTATGCAGCTAAATTAGCTGGTATAGATAAT
TTCAACCAATATATCATTCTTGGTGACGATATAGTCATTAAGAATGATAAGGTTGCCTATTA

TTATATACGTATAATAAATAGACTAGGTGTTGATATATCTCTTACAAAAACACATGTATCAG
AAGATACATATGAATTTGCAAAGAGATGAATAAAGTGTGGAAGAGAAATCACTGGAATAC
CAGTGAGAGGGATTATCCATAATTTTAAAAATATAAATGTAGTATTTACAATACTATATTCA
CATTTTAAAATTAATGGTAATACCTATCTTTCAAAATACAGTTTAGTGGAATCCCTCCGTAG
ATTATATAAAGATTTCTATCTCATTAAAGGAAAGAAAAAATACTTTCCTATACGAAATATG
AAATCTTATATAAACAGACTTCAAACCTTTAGTAATATATTAGATATAACCTTTGGTTATTCT
AATGACCAAAGTATCAGACAAATATTCACTAAAAATATTATTAGTGATTATTATATGATAC
CAACGGCACTAGAGGATAGCCTTCTTGAAATCAAGAAGATCCTTAGTACTGGTCTTGGTAA
GTTACTAAGTTCCAATGTAGGTAAAGTTTCGTCGTGACAGACGAAAATAATTGAAAATTAT
GACGATGAAAATCGTAATAATTTAATATATTATCCAACATTTGTTGGGCTTTACAATTACAT
AAACAATATCAAAACAAGAACAAAAAGATGGAGTGGTTCTGAAGAAATTTCAGAATTAAC
ACAAGATCTTAATGTTATAGATGTTGATAAAGTTTTCAGTAAGGAAAGATCAAAATTTGAT
CATCTCCTTACTATAGGAAAAAGTTTAGAAGTAGGTTTCACTAACATCAATAAAACTGATG
AGATCTATTATGGATCTGCAACAGTTGAATCATCACTGACACCGAAAGGTATGCAGTTATG
ATTCTCGAAATCTATAACAAAAGATGTTATGGATTCAATTATTGAAGGTAAGTGAGAACCT
CCAAAACCTCAAATGTCTTATACAGACATGTGAGAGAGCTTGGCTAAGGGAAAGGTATAG
ACTTGATGGTCTATGACCTCTTGAGCTTGTGTTAATTCACAG

2. Supplementary Figure

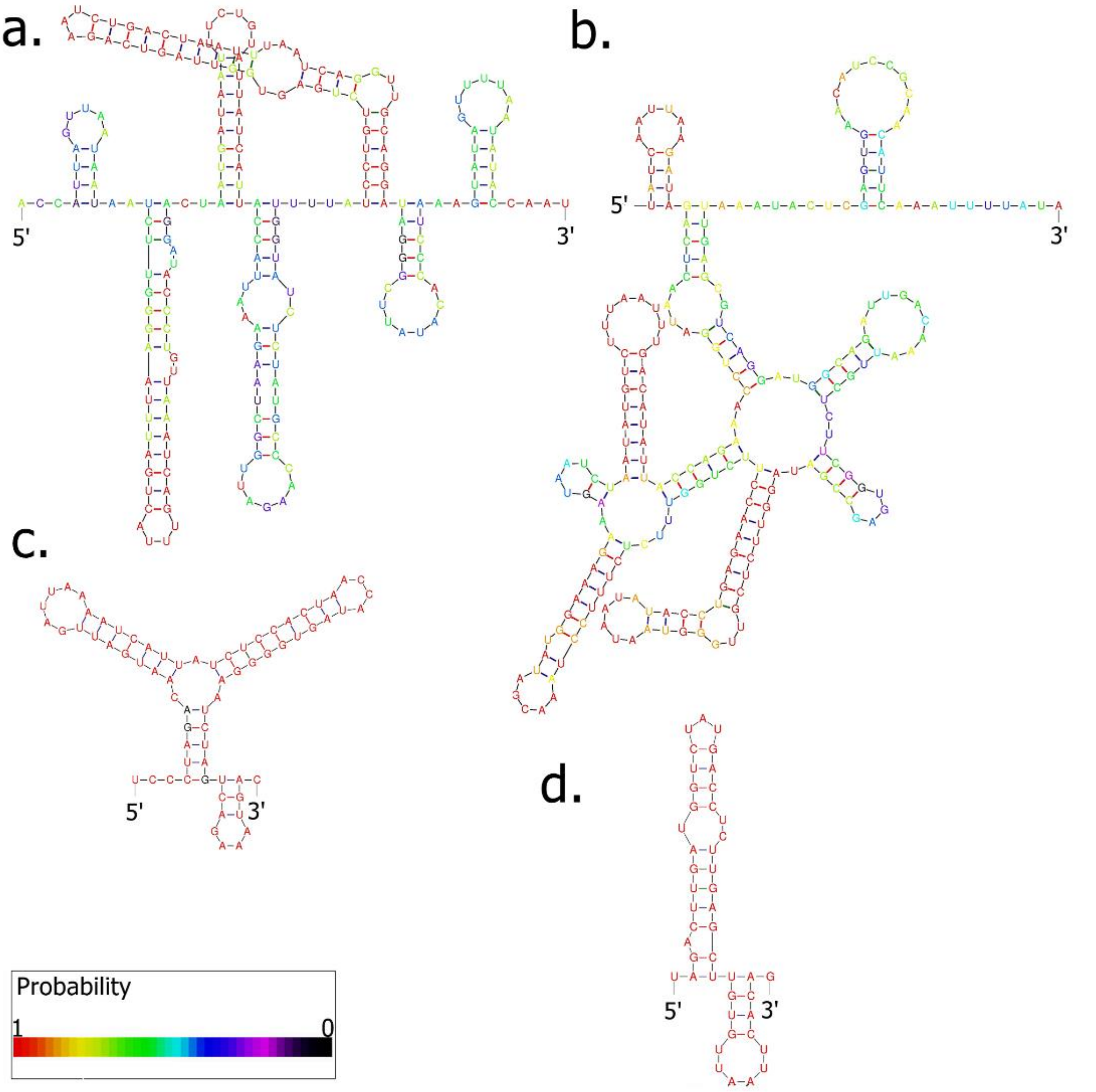

Figure S1. Predicted secondary structures (2-D representation of this self-folding) of the terminal untranslated regions (UTR) of FvMV1 and FaMV1-162. Schemes (a) and (c) represent the 5`-UTR and 3`-UTR of FvMV1 with dG = -82.52 and dG = -29.55, respectively. Schemes (b) and (d) represent the 5`-UTR and 3`-UTR of FaMV1-162 with dG = -73.96 and dG = -13.93, respectively. The +ssRNA molecules were folded, and the free energy was calculated with the RNA Folding Form V 2.3 Energies (MFOLD) program. For these calculations, the following conditions were sectioned: 25ºC, 1M NaCl and 0M divalent ions. The rendering of the structures has been defined with natural angles and annotated using colored base characters, based on p-num information. Colors are ranged from red to black representing the probability as well-determined (1) to poorly determined (0), respectively.

© 2020 by the authors. Submitted for possible open access publication under the terms and conditions of the Creative Commons Attribution (CC BY) license (http://creativecommons.org/licenses/by/4.0/).
